# Causes of disability in one of the largest public universities in Brazil: is there a relationship with employment position?

**DOI:** 10.47626/1679-4435-2023-1174

**Published:** 2024-02-16

**Authors:** Cesar Romaro Pozzobon, Glaucia Maria Moraes de-Oliveira, Gabriel Porto Soares

**Affiliations:** 1 Superintendência de Atenção à Saúde do Trabalhador, Universidade Federal do Rio de Janeiro, Rio de Janeiro, RJ, Brazil; 2 Clínica Médica, Hospital Barra D'Or, Rede D'Or São Luiz, Rio de Janeiro, RJ, Brazil; 3 Programa de Pós-Graduação em Cardiologia, Universidade Federal do Rio de Janeiro, Rio de Janeiro, RJ, Brazil; 4 Curso de Medicina, Universidade de Vassouras, Rio de Janeiro, RJ, Brazil

**Keywords:** government employees, retirement, public sector, universities, servidores públicos, aposentadoria, setor público, universidades

## Abstract

**Introduction:**

Disability retirement is granted to civil servants considered permanently incapable of working. Noncommunicable diseases are the main cause of permanent disability and retirement in Brazil. The Federal University of Rio de Janeiro is one of the largest universities in Brazil, and determining the profile of employees who receive disability pensions at this institution is of great relevance.

**Objectives:**

To describe the profile of university employees who retired due to a disability between 2003 and 2017.

**Methods:**

This cross-sectional study was based on disability retirement records for civil servants. Demographic variables such as sex, age at retirement, and employment position were evaluated.

**Results:**

A total of 630 cases were analyzed, including 334 (53%) full and 296 (47%) proportional retirements; 499 (79.2%) were aged 30 to 59 years at retirement, and 368 (51.4%) were women. The full retirement rate was higher among those with senior level positions (p < 0.001), in older age groups (p < 0.001), and in men (p = 0.012).

**Conclusions:**

Noncommunicable disease was the main cause of retirement. The mean age at permanent disability was early, regardless of sex or retirement type. Permanent disability was more common among employees in positions requiring less education. The disability rate was highest among women.

## Abstract (pt)

**Introdução:**

A aposentadoria por invalidez é o benefício concedido aos servidores públicos quando considerados incapazes de forma permanente para o trabalho. As doenças não comunicáveis são a principal causa de incapacidade permanente e aposentadoria por invalidez no Brasil. A Universidade Federal do Rio de Janeiro é uma das maiores do Brasil, e conhecer o perfil das aposentadorias por invalidez dentro dessa instituição possui grande relevância.

**Objetivos:**

Descrever o perfil das aposentadorias por invalidez na Universidade Federal do Rio de Janeiro no período entre 2003 e 2017.

**Métodos:**

Estudo seccional baseado no registro das aposentadorias por invalidez dos servidores. Foram avaliadas variáveis demográficas como sexo, idade na ocasião da aposentadoria e cargo de ingresso na universidade.

**Resultados:**

Foram analisados 630 casos, com 334 (53%) aposentadorias integrais e 296 (47%) proporcionais. Na ocasião da aposentadoria, 499 (79,2%) servidores apresentavam idade entre 30 e 59 anos e 368 (51,4%) eram do sexo feminino. Foi observado que, nos cargos de nível superior, há maior ocorrência de aposentadorias integrais (p < 0,001 ), assim como nas faixas etárias mais elevadas (p < 0,001 ) e também no sexo masculino (p = 0,012).

**Conclusões:**

As doenças não comunicáveis foram as principais causas das aposentadorias. A média de idade da invalidez permanente foi precoce, independentemente do sexo e do tipo de aposentadoria. Servidores de cargos de menor escolaridade estiveram entre os que mais apresentaram incapacidade permanente. O sexo feminino foi o que mais apresentou invalidez.

## INTRODUCTION

Disability retirement is granted to civil servants who, due to illness or accident and after expert assessment, are considered permanently incapable of working. Disability retirement may be preceded by a leave of ≤24 months. Once this period has expired, employees who cannot resume working or qualify for reassignment will be retired.^1^

Full retirement benefits are granted to employees who have an accident while on duty or develop an occupational illness or other serious illness specified by law. In all other cases, the benefits will be proportional to length of service. Law 8112 (December 11, 1990), which provides for a Single Legal Regime governing federal civil servants, considers the following to be serious illnesses: active tuberculosis, mental illness, multiple sclerosis, malignant neoplasia, blindness after entering public service, leprosy, severe heart disease, Parkinson's disease, irreversible and disabling paralysis, ankylosing spondylitis, severe nephropathy, advanced stages of Paget's disease of bone, and acquired immune deficiency syndrome.^1^ In 2001, radiation contamination and severe liver disease were added to this list through Interministerial Ordinance MPAS/MS 2998 (August 23, 1990).^2^

According to the World Health Organization, the Pan American Health Organization and other authors, noncommunicable diseases, especially cardiovascular disease, musculoskeletal disease, mental disorders, and neoplasms, are the main cause of early disability and disability retirement in most countries in the Americas, including Brazil.^3,4,5^ Noncommunicable diseases typically have multiple etiologies, diverse risk factors, a prolonged clinical course, a non-infectious origin, and are associated with functional disability.^6^

In Brazil, the incidence of disability pensions has increased over the years and has become a relevant problem, having a significant impact on the economically active population.^7^ Approximately 14.5% of all pensions under the General Social Security Program are disability pensions, according to National Social Security Institute estimates. For civil servants with statutory employment contracts, who have a specific pension system under the Single Legal Regime, there is less information, which indicates the importance of research on the topic.^1,8^

The Federal University of Rio de Janeiro (UFRJ), founded in 1792, is one of the largest universities in Brazil, with more than 12,000 employees, including approximately 4000 faculty and 8000 administrators.^9^ There is great socioeconomic and labor diversity among employees due to the UFRJ career plan, with working conditions and salaries differing according to occupation. Some authors postulate that these differences significantly contribute to disability retirement type, making this population of even greater interest.^10^

Thus, understanding the profile of employees who receive disability pensions at this institution is of great relevance on a national level, since it allows reassessment of worker health policies at a time of demographic transition, in which noncommunicable diseases are greatly impacting the work capacity of civil servants.^11^ Hence, this study's objective was to determine the profile of employees who received disability retirement at UFRJ between 2003 and 2017, including their distribution according to sex, age group, and initial position.

## METHODS

This was a cross-sectional study of UFRJ employees who received disability retirement between January 2003 and December 2017. Retirement data were obtained from the minutes of the Official Medical Board and the Medical and Dental Examiner's Office of UFRJ's Department of Occupational Health. All UFRJ employees with a statutory contract who received disability retirement during the study period were included. Retirees who reverted to permanent active status were excluded.

This study was approved by the University of Vassouras Research Ethics Committee (opinion 4,350,685/2020) and the UFRJ Personnel Department (Pró-Reitoria 4).

For the initial data analysis, retirements were classified as full or proportional according to Section I, Article 186, Law 8112/90 and Interministerial Ordinance 2998 (2001). Full retirements were subdivided into 5 groups according to illness type: Group 1, severe heart disease; Group 2, malignant neoplasia, severe liver disease, and severe nephropathy; Group 3, mental illness; Group 4, physical disabilities; and Group 5, leprosy, active tuberculosis, and acquired immunodeficiency syndrome. The following pathologies were included in Group 4: Parkinson's disease, multiple sclerosis, blindness after entering public service, ankylosing spondylitis, irreversible and disabling paralysis, and advanced stages of Paget's disease of bone.

The demographic variables sex, age at retirement, and initial employment position were evaluated in both the inter- and intra-group analyses. Age was subdivided into 3 groups: 30-59 years, 60-64 years, and 65-70 years, based on the minimum and maximum ages provided for in Law No. 8112/90 for the retirement of women and men (60 and 65 years, respectively). Likewise, 3 classes of initial positions were considered based on the career plan for educational administrators at UFRJ: faculty, senior administrators, and junior or entry-level administrators.

Microsoft Excel 16 (Microsoft, Redmond, WA, USA) was used for data collection and IBM SPSS Statistics 24 (IBM, Armonk, NY, USA) was used for statistical analyses. The results were expressed as absolute numbers and percentages for categorical variables and as mean and standard deviation (SD) for numerical variables.

The chi-square test was used to compare categorical variables. The Mann-Whitney test was used to compare age between sexes and within each retirement type, while the Kruskal-Wallis test was used to compare occupations. Univariate and multiple logistic regression models were used to determine the association between demographic data in proportional and full retirement groups. A 95% confidence interval was used, with p-values < 0.05 considered statistically significant.

## RESULTS

**Figure 1.**
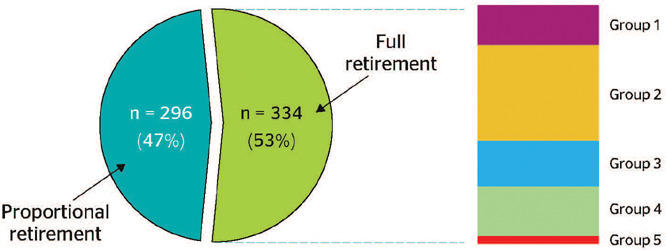
Distribution of retirement pensions among Federal University of Rio de Janeiro employees between 2003 and 2017 according to type and disease group. Group 1: severe heart disease; Group 2: neoplasms, liver disease, or nephropathy; Group 3: mental illness; Group 4: physical disabilities; Group 5: leprosy, AIDS, or tuberculosis.

Of the 700 employees who received disability retirement between January 2003 and December 2017, 70 (10%) to active status and were excluded from the study. Thus, 630 cases were analyzed, of which 334 (53%) were full and 296 (47%) were proportional retirements. Figure 1 shows the distribution of retirement pensions according to type and disease group based on Law 8112/90.

In the initial demographic data analysis for both retirement types, 499 (79.2%) employees retired at 30-59 years of age, 77 (12.2%) at 60-64 years of age, and 54 (8.6%) at 65-70 years of age. A total of 262 (41.6%) men and 368 (51.4%) women received disability retirement. The mean retirement age was 52.9 (SD = 7.8) years: 53.9 (SD = 7.3) years for men and 52.2 (SD = 8.1) years for women. When retirements were compared according to age, sex, and type, disability tended to occur at an earlier age among women who received proportional retirements (p = 0.016) (Table 1).

**Table 1.** Descriptive statistics on the age of Federal University of Rio de Janeiro employees who received disability retirement between 2003 and 2017 according to sex and retirement type

Descriptive statistics of age at retirement	Total	Retirement type					
		Full			Proportional		
		Total	Male	Female	Total	Male	Female
n	630	334	157	177	296	105	191
Mean	52.9	54.4	54.8	54.1	51.2	52.5	50.5
SD	7.8	7.7	7.5	7.8	7.7	6.7	8.1
Minimum	30.0	34.0	37.0	34.0	30.0	39.0	30.0
First quartile	47.0	49.0	49.0	49.0	46.0	48.0	45.0
Median	53.0	54.0	55.0	54.0	51.0	52.0	50.0
Third quartile	58.0	59.0	60.0	59.0	56.0	57.0	56.0
Maximum	70.0	70.0	69.0	70.0	69.0	68.0	69.0
p-value			0.464			0.016	

Initial employment position, age at retirement, and sex were also compared according to retirement type, revealing that full retirement was more common among those in in senior positions (p < O.OOl) and proportional retirement was more common among those in junior or entry-level positions (p < 0.001). Full retirement was more common than proportional retirement among the 60-64 and 65-70 year age groups, (p = 0.012). Finally, there were more full retirements than proportional retirements among men (p = 0.003) (see Table 2).

**Table 2.** Federal University of Rio de Janeiro employees who retired due to disability between 2003 and 2017 according to initial position, age at retirement, sex, and retirement type

Position, retirement age, and sex	Total (n = 630; 100.0 %)		Retirement type				p-value in χ² test
			Full (n = 334; 53.0%)		Proportional (n = 296; 47.0%)		
	n	%	n	%	n	%	
Position							<0.001
Faculty	73	11.6	61	18.2	12	4.0	
Senior administrator	92	14.6	56	16.8	36	12.2	
Junior/entry-level administrator	465	73.8	217	65.0	248	83.8	
Retirement age (years)							0.012						
30-59	499	79.2	251	75.1	248	83.8							
60-64	77	12.2	45	13.5	32	10.8							
65-70	54	8.6	38	11.4	16	5.4							
Sex							0.003
Male	262	41.6	157	47.0	105	35.5	
Female	368	58.4	177	53.0	191	64.5	
Total	630	100.0	334	100.0	296	100.0	

After adjustment in logistic regression models, this association was found to be independent, which corroborated previous findings. Thus, among senior employees, full retirement was more frequent than proportional retirement, especially in faculty positions (adjusted odds ratio [OR_adj_] = 5.04; p < 0.001). Full retirement also occurred at a more advanced age, mainly between 65 and 70 years of age (OR_adj_ = 1.97; p = 0.038) and was more frequent in men (OR_adj_ = 1.51; p = 0.015) (Table 3).

**Table 3.** Logistical models for full retirement (vs proportional) for Federal University of Rio de Janeiro employees who received disability retirement between 2003 and 2017

Position, retirement age, and sex	Univariate		Multivariate	
	OR	p-value	OR_aj_	p-value
Position		<0001		<0.001
Faculty	5.81	<0001	5.04	<0.001
Senior administrators	1.78	0.014	1.82	0.012
Junior/entry-level administrators	1		1	
Retirement age (years)		0.013		0.103
30-59	1		1	
60-64	1.38	0.185	1.19	0.495
65-70	2.34	0.006	1.97	0.038
Sex				
Male	1.61	0.003	1.51	0.015
Female	1		1	

OR = odds ratio; OR_aj_ = adjusted OR.

In the intra-group analysis, full retirements were subdivided according to the diseases specified in Law 8112/90 and were evaluated according to demographic data, as shown in Table 4. Group 2 diseases were more frequent, regardless of the employment position, age at retirement, or sex. There were no Group 5 diseases among faculty or the 65-70 year age group.

**Table 4.** Federal University of Rio de Janeiro employees who received full disability retirement between 2003 and 2017 according to disease group, position, age, and sex

Position, retirement age, and sex	Total		Disease groups for full retirement according to Law 8112/90									
			Group 1: severe heart disease		Group 2: neoplasms, liver disease, or nephropathy		Group 3: mental illness		Group 4: physical disabilities		Group 5: leprosy, AIDS, or tuberculosis	
	n	%	n	%	n	%	n	%	n	%	n	%
Position												
Faculty	61	100.0	7	11.5	33	54.1	12	19.7	9	14.8	0	0.0
Senior administrator	56	100.0	6	10.7	30	53.6	6	10.7	13	23.2	1	1.8
Junior/entry-level administrator	217	100.0	40	18.4	74	34.1	45	20.7	48	22.1	10	4.6
Retirement age (years)												
30-59	251	100.0	40	15.9	104	41.4	49	19.5	49	19.5	9	3.6
60-64	45	100.0	8	17.8	17	37.8	7	15.6	11	24.4	2	4.4
65-70	38	100.0	5	13.2	16	42.1	7	18.4	10	26.3	0	0.0
Sex												
Male	157	100.0	33	21.0	49	31.2	31	19.7	36	22.9	8	5.1
Female	177	100.0	20	11.3	88	49.7	32	18.1	34	19.2	3	1.7
Total	334	100.0	53	15.9	137	41.0	63	18.9	70	21.0	11	3.3

According to analysis of full retirements according to sex and employment position, the mean age at retirement was higher for men than women, but this difference was more pronounced among male faculty members (p = 0.002) than other positions, as shown in Table 5.

**Table 5.** Descriptive statistics on age at full disability retirement among Federal University of Rio de Janeiro employees between 2003 and 2017 according to sex and position

Descriptive statistics for age at retirement	Total	Sex							
		Male				Female			
		Total	Faculty	Senior admin	Junior or entry-level admin	Total	Faculty	Senior admin	Junior or entry-level admin
n	334	157	36	22	99	177	25	34	118
Mean	54.4	54.8	58.6	54.8	53.4	54.1	55.8	52.5	54.2
SD	7.7	7.5	79	7.7	6.9	7.8	7.1	8.7	7.6
Minimum	34.0	37.0	39.0	42.0	37.0	34.0	46.0	34.0	37.0
First quartile	49.0	49.0	54.0	50.0	47.0	49.0	51.0	47.0	49.0
Median	54.0	55.0	58.5	54.5	54.0	54.0	54.0	51.0	54.5
Third quartile	59.0	60.0	65.0	60.0	59.0	59.0	60.0	60.0	58.0
Maximum	70.0	69.0	69.0	69.0	69.0	70.0	69.0	70.0	69.0
p-value		0.002				0.372			

admin. = administrators.

## DISCUSSION

This cross-sectional study investigated the profile of employees who received disability retirement at UFRJ, one of the largest federal universities in Brazil, including distribution by sex, age group, and initial employment position. This study focused on full disability pensions, those resulting from illnesses described in Law 8112/90, since pensions of this type allow full benefits and, often, income tax exemption.

Although previous Brazilian studies have evaluated disability pensions, the majority have been conducted within the scope of the General Social Security Program.^2,3,7,12^ However, studies conducted at federal institutions with specific pension regimes, such as the Single Legal Regime, have had smaller samples or shorter study periods.^13,14,15^

Although not the main objective of this study, the results of the comparison between retirement types were consistent with the literature in that proportional retirements, those resulting from illnesses not foreseen in Law 8112/90, were more common in women and occurred at an earlier age. Similar findings were observed in a study conducted at Londrina State University in 2016,^14^ as well as in other national and international studies.^8,16^ This may be due to the fact that throughout their active lives, in addition to work activities, women also generally assume responsibility for domestic activities, which can increase work overload and health-related problems.

Assessment of retirement type according to the initial employment position showed that more full retirements than proportional retirements occurred among faculty and senior administrators. The flexible work shifts involved in these positions may explain these results. By law, faculty members perform two-thirds of their workload in the classroom and the remainder in scientific production, having the right to 45 vacation days per year, unlike other employees. Thus, these positions may involve less physical and mental stress.^17,18^

It follows that faculty would have a longer active working life and, thus, would take time off only for more serious illnesses, the type responsible for disability and full retirement. This could also apply to senior administrators, who, despite not having the same flexibility in work schedule, have higher salaries than other employees, thus allowing greater access to health care, which could delay the emergence of serious and disabling illnesses.^19,20^

More full retirements occurred between 65 and 70 years of age, which is probably due to the fact that cardiovascular, cerebrovascular, neoplastic, and neurodegenerative diseases increase in direct proportion to age.^21^ However, the number of disability retirements, regardless of type, increased inversely with employee age, as previously reported in a number of studies, thus reinforcing the finding that noncommunicable diseases are removing increasing numbers of civil servants from the work force.^22,23^

Finally, there were more full retirements than proportional retirements among men, although the opposite occurred among women, despite their underrepresentation in this sample. Again, this could be explained by the accumulation of professional and personal roles by women over the years, and could progressively decrease as women gain more authority and appreciation in the professional environment, obtaining equal working hours and salaries, with greater attention paid to their health and quality of life in the workplace.^22,24^

The lack of a control group consisting of employees who voluntarily retired due to length of service was a limitation for certain variables. Another limitation is the scarcity of similar studies on employees governed by the Single Legal Regime, which, although making this study original, limits comparison of its results.

## CONCLUSIONS

The present study identified the profile of employees who received disability retirement at one of the largest public universities in Brazil over a 15-year period. The following conclusions were drawn: noncommunicable diseases were the main cause of retirement; the mean age of retirement for permanent disability was early, regardless of sex or retirement type; disability retirement was more common in positions requiring lower education levels; and that most permanent disability occurred in women.

Understanding the factors involved in disability retirement, including its causes and peculiarities, highlights the need to invest in disease prevention, health promotion, and health education programs to ensure better working conditions and employee health, thus reducing absenteeism and early disability in among federal civil servants.
